# Iron-Doped Monoclinic Strontium Iridate as a Highly Efficient Oxygen Evolution Electrocatalyst in Acidic Media

**DOI:** 10.3390/nano13050797

**Published:** 2023-02-22

**Authors:** Mengjie Li, Jiabao Ding, Tianli Wu, Weifeng Zhang

**Affiliations:** 1 Henan Key Laboratory of Photovoltaic Materials, Henan University, Kaifeng 475004, China; 2 Center for Topological Functional Materials, Henan University, Kaifeng 475004, China

**Keywords:** perovskite oxides, dopant, electrocatalyst, oxygen evolution reaction

## Abstract

Ir-based perovskite oxides are efficient electrocatalysts for anodic oxygen evolution. This work presents a systematic study of the doping effects of Fe on the OER activity of monoclinic SrIrO_3_ to reduce the consumption of Ir. The monoclinic structure of SrIrO_3_ was retained when the Fe/Ir ratio was less than 0.1/0.9. Upon further increases in the Fe/Ir ratio, the structure of SrIrO_3_ changed from a 6H to 3C phase. The SrFe_0.1_Ir_0.9_O_3_ had the highest activity among the investigated catalysts with the lowest overpotential of 238 mV at 10 mA cm^−2^ in 0.1 M HClO_4_ solution, which could be attributed to the oxygen vacancies induced by the Fe dopant and the IrOx formed upon the dissolution of Sr and Fe. The formation of oxygen vacancies and uncoordinated sites at the molecular level may be responsible for the improved performance. This work explored the effect of Fe dopants in boosting the OER activity of SrIrO_3_, thus providing a detailed reference to tune perovskite-based electrocatalyst by Fe for other applications.

## 1. Introduction

The energy crisis is an important issue that needs to be solved urgently in modern society. Energy consumption and overuse of fossil fuels contribute to global warming and climate change. Hydrogen energy carriers offer a potential way to solve this challenge, [1,2] and water splitting can produce hydrogen and uses the hydrogen evolution reaction (HER) at the cathode and oxygen evolution reaction (OER) at the anode [3,4,5]. Unlike the hydrogen reaction at the cathode, the half-reaction at the anode is sluggish due to the multi-proton/electron-coupled process, which usually plays a critical part in the total efficiency of the system [6,7,8]. Hence, substantial efforts have been dedicated to the exploration of efficient OER electrocatalysts to reduce the reaction barrier over the past decade [9,10,11]. Many electrocatalysts based on transition metals are effective for OER under base electrolyte, but only a few of the recently described electrocatalysts have been investigated for utilization in acidic solution due to their extremely corrosive condition [4,12,13]. Ruthenium (Ru)- and iridium (Ir)- based oxides are recognized as state-of-the-art catalysts for OER in acidic media [14]. Though the Ru-based oxides exhibit relatively lower overpotentials than the Ir-based oxides, their stabilities are limited under acidic conditions, [15] and their low abundance and high costs further limit industrialization. Hence, there is an urgent need for high-performance and stable catalysts that consume minimal Ir content.

In recent years, high-entropy materials have been fine-tuned due to their compositional flexibility, and high-entropy mixing provides structural stability under operating conditions. It has been proven to be a unique platform for designing surface composition and active sites, and developing efficient electrocatalysts for water splitting using the synergistic effect of multiple components [16]. Rutile ruthenium oxide (RuO_2_) has strong oxygen-binding ability. Many reports have effectively adjusted the intrinsic electronic structure and the active site of the catalyst to produce defects by doping transition metals in RuO_2_. Moreover, the doped RuO_2_ catalyst, combined with nanostructure and morphology engineering, performs significant enhanced mass activity and stability [17]. Perovskite oxides are widely used in various energy-related electrocatalyst fields due to their lower cost, flexible structure and high catalytic activity. It is an effective way to improve electrocatalytic performance of perovskites by reducing the size of large pieces to the nanoscale [18]. Perovskite-type oxides are usually defined by the formula ABO_3_ and can accommodate a variety of cations, thus allowing the partial substitution of cations at the A sites or B sites. The transition metal at the B site of a perovskite oxide is the main catalytic center, which directly affects the catalytic performance of OER, making it easier to tune the electronic structure of a B-site metal ion of a perovskite oxide because of its unique electronic structures and flexible inter-ionic composition [19,20,21]. Ir-based perovskites, such as the SrIrO_3_, have attracted great attention in studies of OER electrocatalysts due to their low cost and high activities in acidic media [6,7,8,22]. To further reduce the content of Ir in the perovskites and modulate their OER activities, transition metals can be introduced into the B site to boost perovskite performance because the d-orbitals provided by the transition metals favor binding oxygen species with optimal barriers. Moreover, iron (Fe) has played a critical role in the oxygen evolution of Ni-based electrocatalysts in alkaline electrolytes because it has numerous advantages including it being highly abundant, non-poisonous, and low in cost. To the best of our knowledge, there is a lack of systematic exploration of the effect of Fe on the OER activity of monoclinic SrIrO_3_.

Here, we report Fe-doped monoclinic SrIrO_3_ (named SrFe_x_Ir_1-x_O_3_) by a solid-state method at atmospheric pressure. The oxygen vacancies and high oxidation state of the Ir generated by the Fe dopant enhanced the OER activity of monoclinic SrIrO_3_. The SrFe_0.1_Ir_0.9_O_3_ exhibits the best OER activity among the investigated samples, and their mass activity at 1.52 V is 1.69-fold higher than that of SrIrO_3_. During the electrochemical tests, amorphous planes successively form after the leaching of Sr and Fe, thus contributing to enhanced catalytic activity.

## 2. Materials and Methods

### 2.1. Materials Synthesis

The four polycrystalline SrFexIr_1−x_O_3_ samples studied in this work were synthesized by conventional solid-phase methods. Powder precursors were used: strontium carbonate (Aladdin Co., Ltd., Shanghai, China), iridium dioxide (Bairui Co., Ltd., Kunming, Yunnan, China) and ferric oxide (Aladdin, Co., Ltd., Shanghai, China). Four different ratios of SrFe_x_Ir_1−x_O_3_ were prepared by changing the ratio of Ir:Fe using Ir:Fe precursor ratios of 1:0.05, 1:0.1, 1:0.15, and 1:0.2. Stoichiometric strontium precursors were always added in a 1:1 ratio to total B-site precursors. The starting powder reagents were mixed, ground and preheated in air at 600 °C in an alumina crucible for 5 h. Then the obtained powder was pulverized, granulated, and calcined in air at 1100 °C for 24 h.

### 2.2. Electrochemical Measurements

All electrochemical measurements were performed in 0.1 M HClO4 electrolyte. Inks were prepared by 2.5 mg of catalyst powder and 1 mg of carbon black in a liquid solution consisting of 470 μL of isopropanol and 30 μL of Nafion (Sigma-Aldrich, Shanghai, China, a 5% mixture of lower fatty alcohols and water). This ink was sonicated for 1 h immediately before dripping onto a polished glassy carbon electrode with an area of 0.071 cm^2^ to obtain a catalyst loading of 0.85 mg cm^−2^. A three-electrode system was used with a clean platinum sheet counter electrode and a saturated calomel electrode reference electrode. The cyclic voltammetry (CV) test was carried out at 0.1 M HClO_4_ saturated with O_2_ (scan rate 100 mV s^−1^). The linear sweep voltammetry (LSV) test was carried out at a scan rate of 5 mV s^−1^ with 85% iR-drop compensation, sweeping from 1.23 up to 1.59 V vs. RHE for the OER current measurements. To measure stability, chronopotentiometric measurements were made at a constant current density of 10 mA cm^−2^. In the range of 0.4~1.3 V, a cyclic voltammetry (CV) test was performed at a scanning rate of 0.1 V s^−1^ to study the redox behavior of the electrocatalyst. To calculate the effective electrochemical active surface area (ECSA), the double-layer capacitance was measured in a non-faradaic potential range (1.136–1.236 V vs. RHE) by recording the current values as a function of scan rate and the scan rates were 10, 20, 40, 60, 80, and 100 mV s^−1^. The double-layer capacitance (C_dl_) was estimated by plotting the Δj = (j_+_−j_−_)/2 at 1.186 V vs. RHE against the scan rate. The ECSA was estimated according to the following equation:

(1) $$\mathrm{ECSA}=\left(\frac{C_{\mathrm{dl}}}{C_{s}}\right)\;\;\;\left({\mathrm{cm}}^{2}\right)$$

Calibration is performed by using a reversible hydrogen electrode (RHE). First, Pt electrodes were washed with 0.1 M HClO_4_ cycling between −2 and 2 V for 1 h. It is then used as a working electrode and a counter electrode, respectively. Prior to calibration, the electrolyte 0.1 M HClO_4_ should be continuously bubbled H_2_ to saturate it with H_2_. A series of potential control time-ampere curves of 500 s were measured, and the current interconversion between the hydroxide and hydrogen evolution reactions was obtained. The resulting potential is the potential of zero net current. In this work, a potential of zero net current was found at −0.286 V compared to the SCE electrode in 0.1 M HClO_4_ (Figure S7). Therefore, Equation (2) can be used to convert the potential measured relative to SCE to the potential relative to RHE:

(2) $$E_{\mathrm{vs}.\mathrm{RHE}}=E_{\mathrm{vs}.\mathrm{SCE}}+0.286\;V$$

Faraday efficiency is calculated by the ratio of the amount of O_2_ produced in the experiment to the actual value. The O_2_ gas produced by the electrochemical reaction is collected by the drainage method, and then the number of moles of the O_2_ produced is calculated according to the ideal gas law. The theoretical value is determined by assuming that 100% of the current output during the reaction is attributed to OER. According to theoretical considerations, the ratio of the amount of O_2_ to the expected amount of O_2_ is the Faraday efficiency.

The dissolution of catalyst in the OER process was determined by inductively coupled plasma optical emission spectrometry (ICP-OES, iCAP7400, Thermo Fisher, Waltham, MA, USA). The catalyst was loaded onto carbon paper with a catalyst load of about 1 mg cm^−2^ and electrolyzed at 10 mA cm^−2^. A small amount (10 mL) of electrolyte is used to meet the device’s detection limits. Electrolyte was collected after 0.5 h, 1 h, 2 h, 4 h, 6 h, and 8 h electrolysis. At each sampling site, 4 mL electrolyte was removed for ICP measurements and replenished to 10 mL with clean electrolyte before the next sampling cycle. The stability number (S-number) was calculated using the following equation [23]:

(3) $$S_{number}=\frac{n_{O_{2}}}{n_{Ir}}$$

### 2.3. Materials Characterization

Powder X-ray diffraction (XRD) patterns were collected on a Bruker D8 Advance X-ray diffractometer with a Cu Kα radiation source (λ = 1.5418 Å). Transmission electron microscopy (TEM) images were collected on a JEM-F200 (JEOL, Tokyo, Japan). Scanning electron microscopy (SEM) images were studied in a JSM 7001F electron microscope at 10 kV. Energy dispersive X-ray analysis (EDXA) was performed using the EDXA system on a JSM-7610F scanning electron microscope. X-ray photoelectron spectroscopy (XPS) was performed on an AXIS SUPRA+ X-ray photoelectron spectrometer. Avantage software was used for peak fitting. The specific surface area was obtained using nitrogen adsorption on a Besorp-Max II (MicrotracBEL, Tokyo, Japan).

### 2.4. Electrochemical Measurements

Electrochemical workstations (CHI 660E, Chenhua Co., Shanghai, China) were used to analyze the HER polarization curves in 0.5 M H_2_SO_4_ at room temperature. A saturated calomel electrode (SCE) and graphite rod were used as the reference electrode and counter electrode, respectively. The SCE electrode was calibrated relative to the reversible hydrogen electrode (RHE). Linear sweep voltammetry (LSV) data were collected at a scanning rate of 2 mV s^−1^. The time dependence of the catalytic current during the electrolysis of the catalyst in 0.5 M H_2_SO_4_ was measured at potentiostatic voltage. 

## 3. Results and Discussion

**Figure 1:**
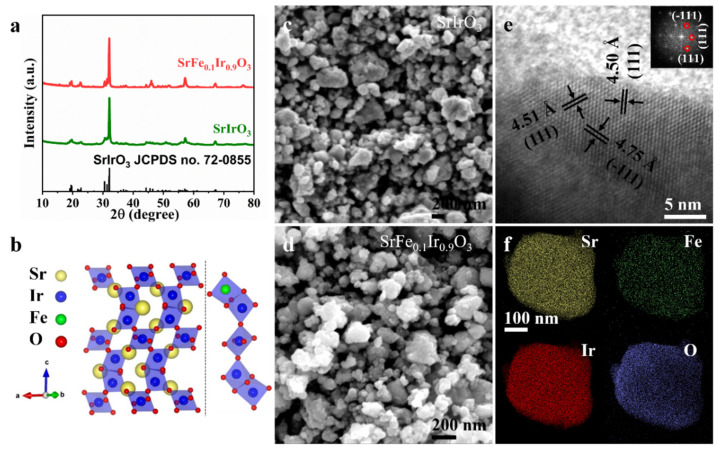
(**a**) XRD patterns of SrFe_0.1_Ir_0.9_O_3_ and SrIrO_3_. (**b**) Crystal structure of SrIrO_3_ and the proposed Fe-doped local connection pattern of IrO_6_ octahedra in monoclinic SrFe_0.1_Ir_0.9_O_3_. (**c**) SrIrO_3_ and (**d**) SrFe_0.1_Ir_0.9_O_3_. (**e**) HRTEM image captured from SrFe_0.1_Ir_0.9_O_3_. Inset: the corresponding fast Fourier transform image. (**f**) The elemental mapping images of a typical nanoparticle of SrFe_0.1_Ir_0.9_O_3_.

Here, the Fe-doped monoclinic SrIrO_3_ with different doping amounts was synthesized via a solid-state method at atmospheric pressure and named SrFe_x_Ir_1-x_O_3_. The SrFe_0.1_Ir_0.9_O_3_ had the best catalytic activity for OER in 0.1 M HClO_4_. The structures and morphologies of the SrFe_0.1_Ir_0.9_O_3_ and SrIrO_3_ were initially characterized by XRD, SEM, and TEM measurements. Figure 1a shows the XRD patterns of SrIrO_3_ and SrFe_0.1_Ir_0.9_O_3_. The diffraction peaks of SrFe_0.1_Ir_0.9_O_3_ are similar to those of SrIrO_3_ where the clear diffraction peaks correspond to lattice planes of monoclinic SrIrO_3_ (JCPDS card no.72-0855), thus revealing a crystalline structure of SrIrO_3_ after doping with Fe. Further comparisons of the XRD patterns show that the diffraction peaks of SrFe_0.1_Ir_0.9_O_3_ shift to smaller diffraction locations than SrIrO_3_, thus demonstrating a slight expansion of the perovskite framework after Fe doping. This phenomenon can be explained by the fact that the radius of Fe^3+^ ions (65 pm) is larger than that of Ir^4+^ ions (63 pm). Figure S1 shows that the XRD pattern of SrFe_0.05_Ir_0.95_O3 has a similar diffraction peak as SrFe_0.1_Ir_0.9_O_3_ and SrIrO_3_, which exhibits less of a shift toward the lower position of diffraction peaks than SrFe_0.1_Ir_0.9_O_3_ versus SrIrO_3_. When the Fe/Ir ratio increased to 0.15/0.85 and 0.2/0.8, the locations of the main peaks showed a greater shift toward lower angles versus SrFe_0.1_Ir_0.9_O_3_.

The crystal structure gradually transforms from a 6H phase to a 3C phase perovskite structure upon a gradual increase in the Fe content (Figure S1), which is consistent with the literature [24]. The left part of Figure 1b shows the typical crystal structure of monoclinic SrIrO_3_ that consists of alternating face-sharing IrO_6_ octahedra dimers and corner-sharing IrO_6_ octahedra along the c-axis. Previous studies show that the Ir-O bond in the coplanar IrO_6_ octahedral dimer is significantly weaker than that in the angle-sharing isolated octahedron. Fe atoms are more inclined to substitute the Ir atoms on the IrO_6_ octahedral dimer shared by the faces (right part in Figure 1b) [25]. The higher valence state of Ir is increased by the redistribution of electrons from Ir to Fe atoms due to the large number of Fe atoms in the crystal lattice. Different Fe contents have a great influence on the Ir-O bonding electron environment, and the electron orbital hybridization is more complicated. As the iron content increases, lattice distortion which leads to the phase transitions would occur violently in polygon octahedral dimers. The inherent electronic structure and the high valence state of Ir atom contribute to its high catalytic activity and good stability, respectively, thereby optimizing the water dissociation kinetics [26]. The SEM images of SrIrO_3_ and SrFe_0.1_Ir_0.9_O_3_ show that both SrIrO_3_ and SrFe_0.1_Ir_0.9_O_3_ are composed of granular, micron-sized particles, and the size of SrFe_0.1_Ir_0.9_O_3_ nanoparticles become larger (Figure 1c,d). The corresponding Brunauer–Emmett–Teller (BET) surface areas of the above two samples are also provided. SrFe_0.1_Ir_0.9_O_3_ has a lower BET value due to its larger particle size than SrIrO_3_ (Figure S2). The atomic ratios of SrIrO_3_ and SrFe_0.1_Ir_0.9_O_3_ were collected by energy dispersive X-ray spectroscopy (EDX), thus showing the existence of the corresponding elements (Figure S3). Figure 1e shows a high-resolution TEM image of an SrFe_0.1_Ir_0.9_O_3_ nanoparticle. The top-right corner is the corresponding fast Fourier transform (FFT) image. Three groups of lattice fringes with interplanar distances of 4.50 Å, 4.51 Å, and 4.75 Å correspond to the (111), (111), and (−111) crystallographic planes of SrFe_0.1_Ir_0.9_O_3_, respectively. Figure 1f shows the elemental mapping images of SrFe_0.1_Ir_0.9_O_3_ of a representative nanoparticle including even distributions of Sr, Fe, Ir, and O via TEM and high-angle annular dark-field scanning TEM. The elemental distribution of SrIrO_3_ is shown in Figure S4.

**Figure 2:**
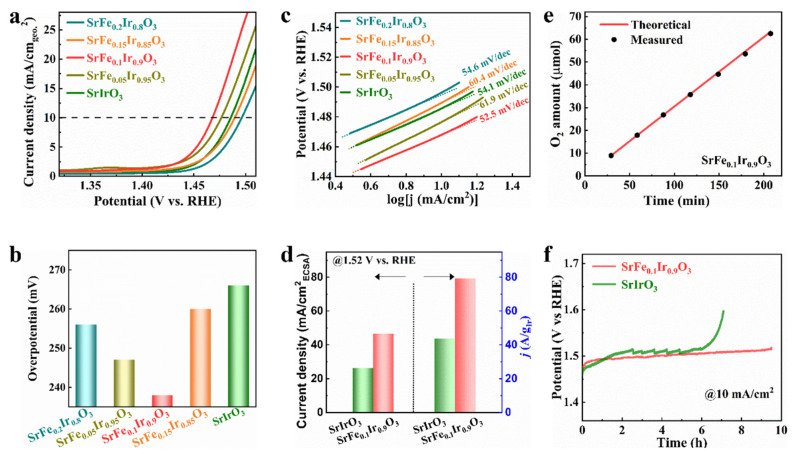
(**a**) Polarization curves of SrIrO_3_ and SrFe_x_Ir_1-x_O_3_ normalized by geometric area in 0.1 M HClO_4_ solution with 85% IR compensations. (**b**) Overpotentials of SrIrO_3_ and SrFe_x_Ir_1-x_O_3_ at 10 mA cm^−2^ in 0.1 M HClO_4_ solution. (**c**) The Tafel slope of the samples in (**a**). (**d**) ECSA and iridium amount normalized current density of SrIrO_3_ and SrFe_0.1_Ir_0.9_O_3_ at 1.52 V. (**e**) The amount of O_2_ theoretically calculated and experimentally measured in the presence of SrFe_0.1_Ir_0.9_O_3_ versus time at a current density of mA cm^−2^. (**f**) Chronopotentiometric curve of SrFe_0.1_Ir_0.9_O_3_ and SrIrO_3_ in 0.1 M HClO_4_ solution at 10 mA cm^−2^ for OER.

To investigate the modulation effect of Fe on the OER activity of SrIrO_3_, the electrocatalytic OER performance of SrFe_x_Ir_1-x_O_3_ was evaluated upon comparison with pristine SrIrO_3_. The electrochemical measurements of SrIrO3 and SrFe_x_Ir_1-x_O_3_ samples were evaluated in 0.1 M HClO_4_ at room temperature using a three-electrode system. Figure 2a shows the 85%IR-corrected polarization curves of SrFe_x_Ir_1-x_O_3_ and SrIrO_3_ normalized by geometric area at a scan rate of 5 mV s^−1^. Versus SrIrO_3_, the SrFe_0.1_Ir_0.9_O_3_ and SrFe_0.05_Ir_0.85_O_3_ showed higher catalytic activities than that of SrIrO_3_. At a current density of 10 mA cm^−2^, the overpotential of SrFe_0.1_Ir_0.9_O_3_ is 238 mV, which is much lower than that of SrIrO_3_ (256 mV) (Figure 2b). The Tafel slopes of the samples derived from the polarization curves are shown in Figure 2c and show the lowest Tafel slope of SrFe_0.1_Ir_0.9_O_3_ (52.5 mV dec^−1^) among the investigated catalysts. The SrFe_0.1_Ir_0.9_O_3_ exhibits a low overpotential and Tafel slope, which are even lower than most of the perovskite oxides (Table S1). The exchange current densities of SrFe_x_Ir_1-x_O_3_ and SrIrO_3_ also confirm the highest activity of SrFe_0.1_Ir_0.9_O_3_ (Figure S5). The currents were normalized with the mass of Ir to carefully evaluate the performance of SrFe_0.1_Ir_0.9_O_3_. The results show that SrFe_0.1_Ir_0.9_O_3_ has higher activity than SrIrO_3_ (Figure 2d). It is clear that the estimated mass activity of SrFe_0.1_Ir_0.9_O_3_ at an overpotential of 290 mV is close to 79.1 A g_Ir_^−1^. This value is 1.69-fold higher than that of SrIrO_3_ (46.6 A g_Ir_^−1^). The electrochemical surface area (ECSA) of the samples was estimated from the double-layer capacitance. The ECSA-normalized current density of SrFe_0.1_Ir_0.9_O_3_ and SrIrO_3_ were also evaluated (Figure 2d). The ECSA-normalized current of SrFe_0.1_Ir_0.9_O_3_ at 1.52 V (vs. RHE) is 1.67-fold higher than that of SrIrO_3_, thus indicating the better activity of SrFe_0.1_Ir_0.9_O_3_ than that of SrIrO_3_. To further evaluate the intrinsic activity of catalysts, the currents were normalized by the BET surface areas illustrating the higher catalytic activity of SrFe_0.1_Ir_0.9_O_3_ than SrIrO_3_ (Figure S6). The Faradaic efficiency of oxygen evolution for SrFe_0.1_Ir_0.9_O_3_ (Figure 2e) illustrates the correlation between time and oxygen production at a current of 10 mA where almost all electrons were transferred toward oxygen evolution. The stability of SrFe_0.1_Ir_0.9_O_3_ and SrIrO_3_ was further examined by galvanostatic measurements. Figure 2f shows the slight potential change during the entire period for SrFe_0.1_Ir_0.9_O_3_ at a geometrical current density of 10 mA cm^−2^, thus illustrating the high stability of SrFe_0.1_Ir_0.9_O_3_.

**Figure 3:**
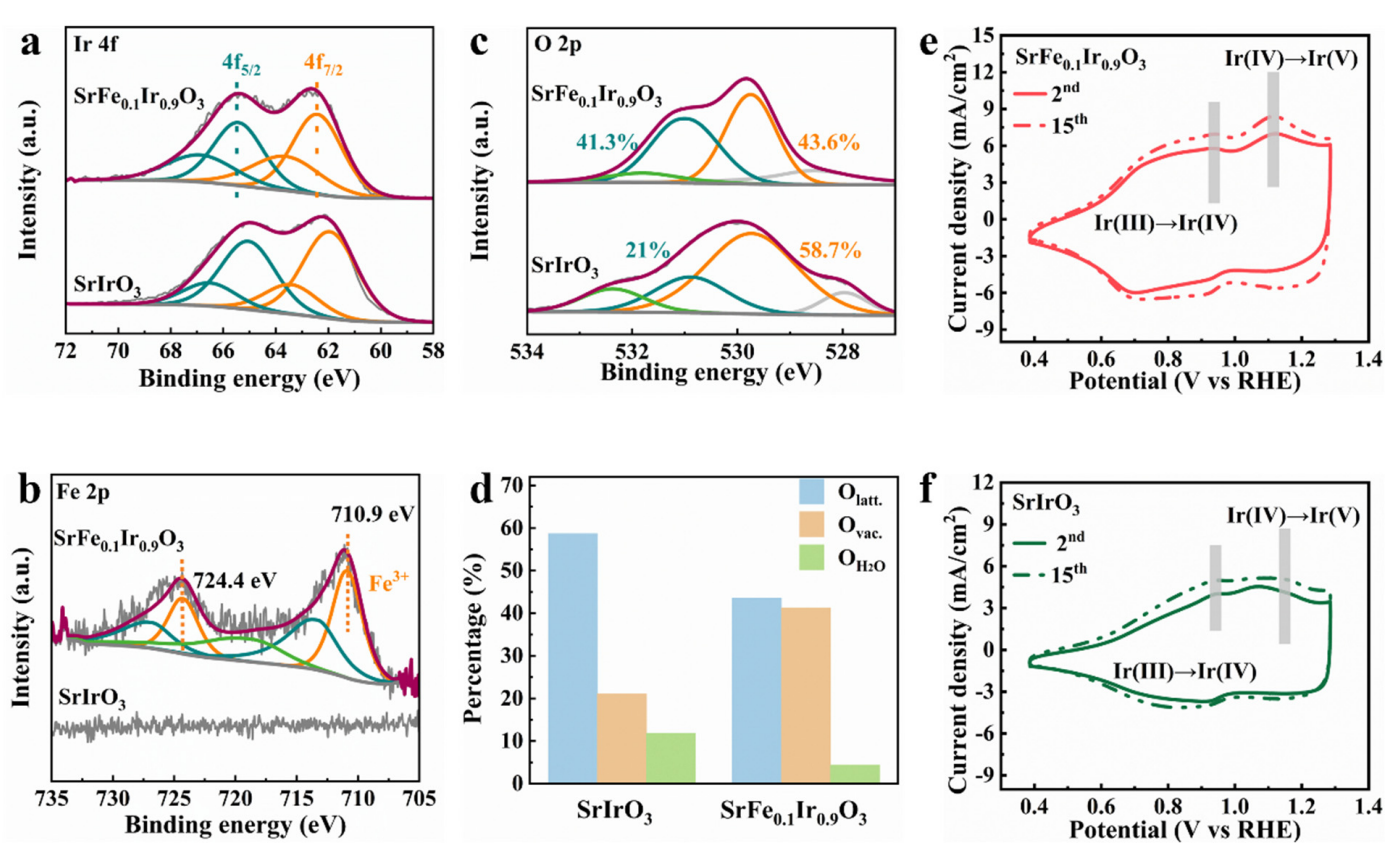
(**a**) Ir 4f, (**b**) Fe 2p, and (**c**) O 2p XPS spectra of SrIrO_3_ and SrFe_0.1_Ir_0.9_O_3_. (**d**) Result of O 1s orbital XPS spectra of SrIrO_3_ and SrFe_0.1_Ir_0.9_O_3_. The cyclic voltammetry (CV) curves recorded at the 2nd and 15th cycles for (**e**) SrIrO_3_ and (**f**) SrFe_0.1_Ir_0.9_O_3_.

The XPS measurements were performed to investigate the chemical states of SrFe_0.1_Ir_0.9_O_3_ and SrIrO_3_. Compared to the Ir 4f XPS spectrum of SrIrO_3_, the binding energies (62.5 eV and 65.5 eV) corresponding to Ir 4f_7/2_ and Ir 4f_5/2_ for the SrFe_0.1_Ir_0.9_O_3_ are at higher values, thus suggesting that the average oxidation state of Ir in SrFe_0.1_Ir_0.9_O_3_ is higher than 4^+^ and regulates the charge caused by the introduction of Fe (Figure 3a). Figure 3b shows the Fe 2p XPS spectra of SrIrO_3_ and SrFe_0.1_Ir_0.9_O_3_. The intensity of the Fe 2p peak is very weak, thus indicating a lower Fe content on the surface of SrFe_0.1_Ir_0.9_O_3_. The two satellite peaks of high-resolution Fe 2p XPS at 710.9 eV and 724.4 eV can be assigned to Fe^3+^. Figure 3c shows that the O 1s XPS spectra of SrIrO_3_ and SrFe_0.1_Ir_0.9_O_3_ are divided into four peaks: lattice oxygen (528.5 eV, 529.7 eV), oxygen vacancy (530.95 eV), and adsorbed water (532.35 eV) [27,28]. According to the literature, higher vacancy concentrations and oxidation states of Ir result in more electrophilic catalysts, thus increasing the deprotonation rate to accelerate the OER reaction [29].

Figure 3d shows the content of different oxygen species in which the content of oxygen vacancies or hydroxyl groups in SrFe_0.1_Ir_0.9_O_3_ is about 20% higher than that of SrIrO3, thus facilitating oxygen species absorption/desorption [30,31,32]. For some compounds, these vacancies are related to the overall catalytic performance. For instance, oxygen vacancies appear when the host and guest atoms have different oxidation states in SrFeO_3_ [33]. Most perovskite materials have a propensity for oxygen vacancies due to solid-phase methods at high temperatures, which leads to the distortion of the oxygen octahedron [34]. The oxygen deficiency is closely related to the bond covalency of M−O and catalytic activity. From the perspective of the adsorption evolution mechanism, the increase in covalency leads to an increase in the electron density of the Ir site and a decrease in the electrostatic interaction with the oxygen intermediate species, thus weakening the binding energy of Ir to the oxygen intermediate species [35,36,37].

Similarly, the Ir−O bond length shortens as the covalent degree increases, which is favorable for the formation of electrophilic oxygen intermediates. Here, the electrophilicity of the oxygen intermediate species is also enhanced and can facilitate the nucleophilic attack of water molecules. Upon the introduction of oxygen vacancies, the valence state of metal ions changes, thus forming more covalent Fe−O bonds [38,39]. Considering that the Ir species with different oxidation states are highly correlated with electrocatalytic activity, we investigated the redox behavior of SrFe_0.1_Ir_0.9_O_3_ and SrIrO_3_ by cyclic voltammetry cycles. Figure 3e,f show that their 2nd and 15th redox curves were collected. Two oxidation peaks can be observed in their CV plots, thus representing Ir^3+^/Ir^4+^ and Ir^4+^/Ir^5+^, respectively. The redox of SrIrO_3_ is mainly Ir^3+^/Ir^4+^. However, Ir^4+^ is easily oxidized to Ir^5+^ for SrFe_0.1_Ir_0.9_O_3_. Ir^5+^ can serve as an active intermediate during the OER process: it increases the OER activity. Therefore, the good activity of SrFe_0.1_Ir_0.9_O_3_ can be attributed to the easier generation of the active intermediate Ir^5+^. The fitting results of SrFe_0.1_Ir_0.9_O_3_ and SrIrO_3_ are listed in Table S2.

**Figure 4:**
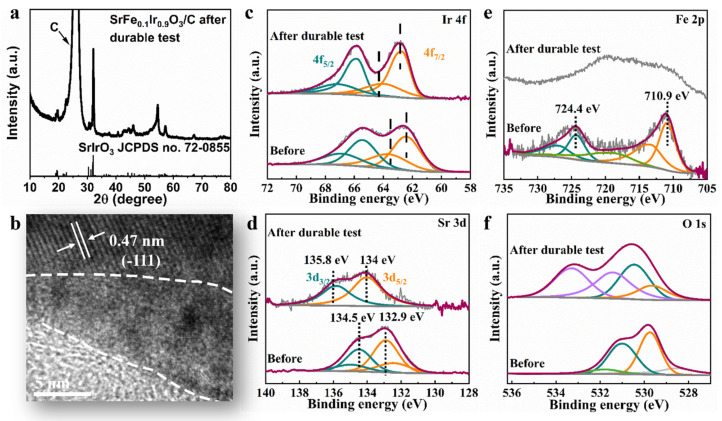
(**a**) XRD pattern of SrFe_0.1_Ir_0.9_O_3_/C after a 10 h durability test, (**b**) HRTEM image of SrFe_0.1_Ir_0.9_O_3_ after a 10 h durability test. (**c**) Ir 4f, (**d**) Sr 3d, (**e**) Fe 2p, and (**f**) O 2p XPS spectra of SrFe_0.1_Ir_0.9_O_3_ before and after 10 h durability test.

Next, to study the stability of the catalyst, the structure of SrFe_0.1_Ir_0.9_O_3_ was investigated by XRD, HRTEM, and XPS after a 10 h durability test. The XRD pattern of SrFe_0.1_Ir_0.9_O_3_ after the stability test has similar diffraction peaks as the SrFe_0.1_Ir_0.9_O_3_ before testing, thus revealing that it retains the main structure (Figure 4a). The HRTEM image captured from SrFe_0.1_Ir_0.9_O_3_ after durability measurements indicates that the surface structure reconstruction occurs during electrochemical processes. The surface region with a depth of about 5–7 nm becomes amorphous, while the body of the SrFe_0.1_Ir_0.9_O_3_ retains its initial crystalline structure. This confirms the XRD data. The cation leaching amount in OER process was quantitatively determined by inductively coupled plasma atomic emission spectrometry (ICP−OES) [40]. As shown in Figure S8, for SrFe_0.1_Ir_0.9_O_3_, the amount of Sr and Fe in the electrolyte was detected to increase for a short time and then level off. The existence of the amorphous state on the surface indicates that a large amount of Sr and Fe dissolved into the solution, and IrO_x_ formed on the surface. The corrosion of Sr and Fe exposes more active sites on the surface of the catalyst, which further enhances its catalytic activity. Figure 4c is the Ir 4f XPS spectrum of SrFe_0.1_Ir_0.9_O_3_ before and after durability testing, thus showing the positive shift of binding energies by approximately 0.4 eV after the stability test, which can be attributed to the formation of high-valence Ir.

The fitting parameters for the Ir 4f XPS spectrum of SrFe_0.1_Ir_0.9_O_3_ are listed in Supplementary Table S3. The Ir 4f peak from the second doublet has an energy shift of 1.6 eV, which is much larger than that observed in the pristine sample (~1.0 eV). This indicates that the surface structure of IrO_6_ becomes more complex. Figure 4d shows the Sr 3d XPS data for SrFe_0.1_Ir_0.9_O_3_ initially and after the durability testing, thus revealing a significant reduction in the Sr of SrFe_0.1_Ir_0.9_O_3_ due to the leaching of Sr from the surface into the electrolyte. The peak positions at the binding energies of 132.9 eV and 134.5 eV are attributed to the lattice Sr in the perovskite. After the durability testing, part of the lattice Sr was converted to surface Sr.

Figure 4e shows that the Fe 2p XPS spectrum has peaks at 710.9 and 724.4 eV, which can be assigned to Fe^3+^. After the OER test, the signal of Fe^3+^ is almost invisible, thus indicating the dissolution of Fe into the electrolyte. Similarly, the positive shift of binding energies is observed in the O 1s XPS spectrum of SrFe_0.1_Ir_0.9_O_3_ after durability testing (Figure 4f). The dissolution of the non-precious metal Fe and the precipitation of Sr introduces a large number of cationic vacancies. When cationic vacancies exist, the content of oxygen species is greatly increased to confirm a charge balance and promote nucleophilic attack of water and the formation of OOH* species [32]. The coordination of oxygen is unsaturated due to the existence of surface defects, which is easy to combine with protons to form surface hydroxyl species [41]. Hydroxyl species are active intermediates on the active sites, and the increase in number of active sites on the catalyst surface further improves the oxygen evolution activity of the samples.

## 4. Conclusions

In this study, it was found that Fe-doped monoclinic SrIrO_3_ with Fe/Ir ratios of 0.05/0.95, 0.1/0.9, 0.15/0.85, and 0.2/0.8 were successfully synthesized by a solid-phase reaction. When the Fe/Ir ratio was 0.1/0.9, the monoclinic structure was retained after Fe doping. Further increases in the Fe/Ir ratio to 0.15/0.85 and 0.2/0.8 led to an SrIrO_3_ change from 6H to 3C. The SrFe_0.1_Ir_0.9_O_3_ exhibited the best activity for OER among the investigated samples with an overpotential of 238 mV at 10 mA cm^−2^, which was much lower than that of SrIrO_3_. The oxygen vacancies induced by the Fe dopants contributed to the enhanced OER activity of SrFe_0.1_Ir_0.9_O_3_. The SrFe_0.1_Ir_0.9_O_3_ catalyst has high activity and good stability, and this work provides a reference for the design of non-noble metal-doped high-efficiency acid water oxidation electrocatalysts to further improve the utilization efficiency of precious metal iridium.

## Data Availability

The data that support the findings of this study are available from the corresponding authors upon reasonable request.

## Supplementary Materials

The following supporting information can be downloaded at: https://www.mdpi.com/article/10.3390/nano13050797/s1. Figure S1: XRD patterns of SrIrO_3_ and SrFe_x_Ir_1-x_O_3_; Figure S2: N_2_ adsorption-desorption isotherms of (**a**) SrIrO_3_ and (**b**) SrFe_0.1_Ir_0.9_O_3_. The graph provides the BET surface area; Figure S3: Energy Dispersive X-ray Spectroscopy (EDX) Study of (**a**) SrIrO_3_ (**b**) and SrFe_0.1_Ir_0.9_O_3_; Figure S4: The corresponding elemental mapping image of SrIrO_3_ (scale bar, 100 nm); Figure S5: Exchange current density of SrIrO_3_ and SrFe_x_Ir_1-x_O_3_ at 10 mA/cm^2^_geo_ in 0.1 M HClO_4_ solution; Figure S6: Comparisons of current densities normalized by BET surface areas for SrIrO_3_ and SrFe_0.1_Ir_0.9_O_3_; Figure S7: The current as a function of the applied potentials for the calibration of SCE reference electrode; Figure S8: Contents of leached metals in the electrolyte in the presence of SrFe_0.1_Ir_0.9_O_3_ during 8 h long electrocatalysis. Table S1: Comparison of OER activities for the catalysts in acid media; Table S2: XPS fit parameters for Ir 4f of SrFe_0.1_Ir_0.9_O_3_ and SrIrO_3_; Table S3: Approximate XPS peak positions and full width half maxes(FWHM) for SrFe_0.1_Ir_0.9_O_3_ after 10 h stability measurement; Table S4: Stability number (S-number) of different catalysts. Refs. [22,24,32,42,43,44,45,46,47,48,49,50] are cited in the supplementary materials.
