# Supplementary material for: Iron-Doped Monoclinic Strontium Iridate as a Highly Efficient Oxygen Evolution Electrocatalyst in Acidic Media
Source: Nanomaterials (Basel). 2023 Feb 22;13(5):797. doi: 10.3390/nano13050797 (PMC10005387; doi:10.3390/nano13050797)
Supplement: Supplementary file 1 [file nanomaterials-13-00797-s001.zip › nanomaterials-2220148-supplementary.pdf]

nanomaterials

Supplementary Materials

Iron-Doped Monoclinic Strontium Iridate as a Highly Efficient Oxygen Evolution Electrocatalyst in Acidic Media

Mengjie Li [1], Jiabao Ding [1], Tianli Wu [1,*] and Weifeng Zhang [1,2,*]

1 Henan Key Laboratory of Photovoltaic Materials, Henan University, Kaifeng 475004, China
2 Center for Topological Functional Materials, Henan University, Kaifeng 475004, China
* Correspondence: tianliwu@henu.edu.cn (T.W.); wfzhang@henu.edu.cn (W.Z.)

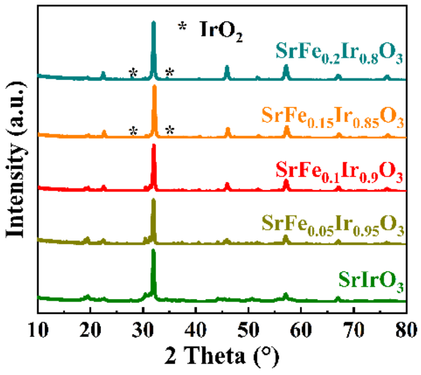

Figure S1. XRD patterns of $SrIrO_3$ and $SrFe_xIr_{1-x}O_3$.

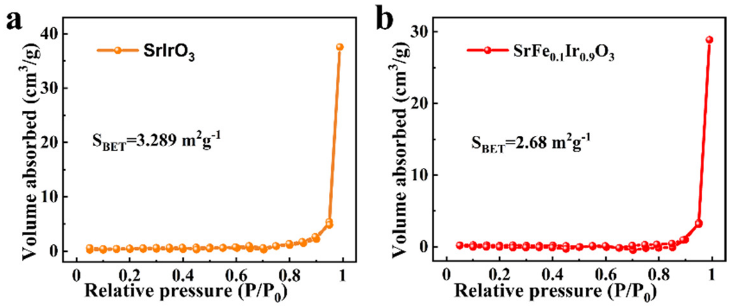

Figure S2. N_2 adsorption-desorption isotherms of (**a**) $SrIrO_3$ and (**b**) $SrFe_{0.1}Ir_{0.9}O_3$. The graph provides the BET surface area.

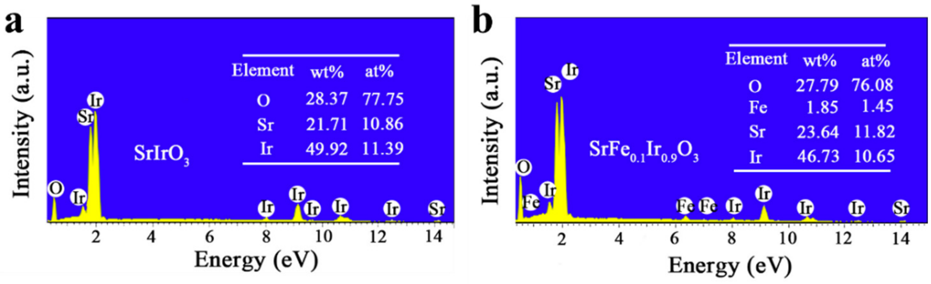

Figure S3. Energy Dispersive X-ray Spectroscopy (EDX) Study of (**a**) $SrIrO_3$ (**b**) and $SrFe_{0.1}Ir_{0.9}O_3$.

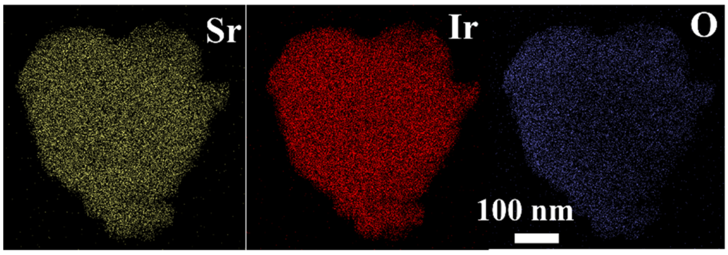

Figure S4. The corresponding elemental mapping image of $SrIrO_3$ (scale bar, 100 nm).

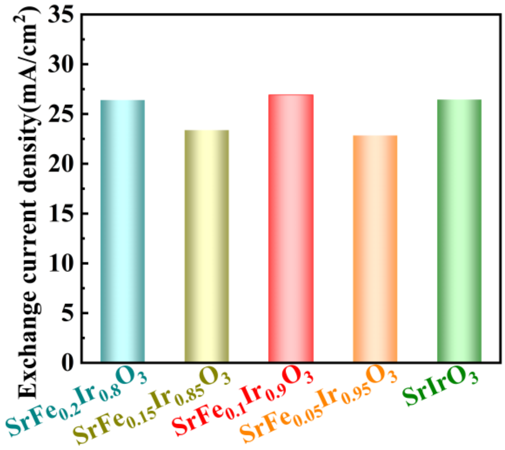

Figure S5. Exchange current density of $SrIrO_3$ and $SrFe_xIr_{1-x}O_3$ at 10 mA/cm^2_{geo} in 0.1 M $HClO_4$ solution.

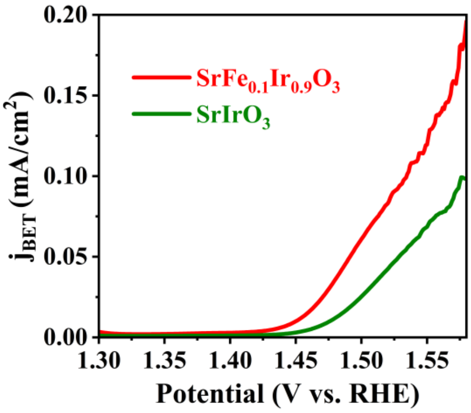

Figure S6. Comparisons of current densities normalized by BET surface areas for $SrIrO_3$ and $SrFe_{0.1}Ir_{0.9}O_3$.

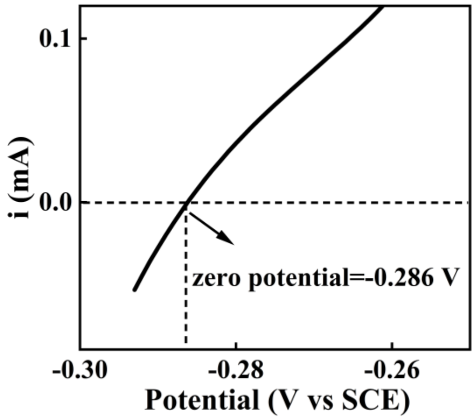

Figure S7. The current as a function of the applied potentials for the calibration of SCE reference electrode.

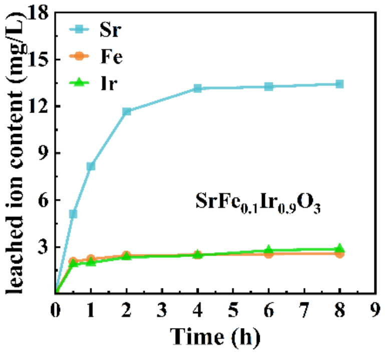

Figure S8. Contents of leached metals in the electrolyte in the presence of $SrFe_{0.1}Ir_{0.9}O_3$ during 8 h long electrocatalysis.

Table S1. Comparison of OER activities for the catalysts in acid media.

Catalyst	Electrolyte	Overpotential (mV) at 10 mA/cm^2	Tafel slope (mV/dec)	Reference
$SrFe_{0.1}Ir_{0.9}O_3$	0.1 M $HClO_4$	238	50.9	This work
6H-$SrIrO_3$	0.1 M $HClO_4$	260	54.2	This work
$Ba_4PrIr_3O_{12}$	0.1 M $HClO_4$	278		[1]
Ba_2PrIrO_6	0.1 M $HClO_4$	400	55	[2]
Sr_2FeIrO_6	0.1 M $HClO_4$	420	90	[3]
Sr_2CoIrO_6	0.1 M $HClO_4$	305	52	[3]
Sr_2NiIrO_6	0.1 M $HClO_4$	295	48	[3]
$SrIr_{0.8}Zn_{0.2}O_3$	0.1 M $HClO_4$	300		[4]
$SrCo_{0.9}Ir_{0.1}O_{3-\delta}$	0.1 M $HClO_4$	320		[5]
La_2LiIrO_6	0.5 M H_2SO_4	300	50	[6]
$IrO_x/SrIrO_3$	0.5 M H_2SO_4	270~290		[7]
$Pr_2Ir_2O_7$	0.1 M $HClO_4$	300		[8]
$Nd_2Ir_2O_7$	0.1 M $HClO_4$	325		[8]
Co doped $SrIrO_3$	0.1 M $HClO_4$	235	51.8	[9]
$SrTi_{0.67}Ir_{0.33}O_3$	0.1 M $HClO_4$	247		[10]
$SrZrO_3$-$SrIrO_3$	0.1 M $HClO_4$	240		[11]
$CaCuRuO_3$	0.5 M H_2SO_4	171	40	[12]

Table S2. XPS fit parameters for Ir 4f of $SrFe_{0.1}Ir_{0.9}O_3$ and $SrIrO_3$.

		$4f_{5/2}$	$4f_{7/2}$	$4f_{5/2}$sat	$4f_{7/2}$sat
$SrFe_{0.1}Ir_{0.9}O_3$	Binding energy (eV)	65.42	62.34	66.88	63.81
	FWHM (eV)	2.32	2.32	3.1	3.1
$SrIrO_3$	Binding energy (eV)	65.04	61.94	66.47	63.37
	FWHM (eV)	1.46	1.42	1.72	1.72

Table S3. Approximate XPS peak positions and full width half maxes(FWHM) for $SrFe_{0.1}Ir_{0.9}O_3$ after 10 h stability measurement.

After OER test	Ir $4f_{5/2}$	Ir $4f_{7/2}$	Ir $4f_{5/2}$sat	Ir $4f_{7/2}$sat
Binding energy (eV)	65.84	62.74	67.5	64.28
FWHM (eV)	1.38	1.38	2.8	2.8

Table S4. Stability number (S-number) of different catalysts.

Catalysts	S-number after 8 h
$SrFe_{0.1}Ir_{0.9}O_3$	25260
$SrIrO_3$	20368

References

1. Gao R, Gao, R.; Zhang, Q.; Chen, H.; Chu, X.; Li, G, D.; Zou, X. Efficient acidic oxygen evolution reaction electrocatalyzed by iridium-based 12L-perovskites comprising trinuclear face-shared IrO_6 octahedral strings. *J. Energy Chem.* **2020**, *47*, 291-298.
2. Diaz-Morales, O.; Raaijman, S.; Kortlever, R.; Kooyman, P. J.; Wezendonk, T.; Gascon, J.; Koper, M. T. Iridium-based double perovskites for efficient water oxidation in acid media. *Nat. Commun*. **2016**, *7,* 1-6.
3. Retuerto, M.; Pascual, L.; Piqué, O.; Kayser, P.; Salam, M. A.; Mokhtar, M.; Rojas, S. How oxidation state and lattice distortion influence the oxygen evolution activity in acid of iridium double perovskites. *J. Mater. Chem. A* **2021**, *9,* 2980-2990.
4. Edgington, J.; Schweitzer, N.; Alayoglu, S.; Seitz, L. C. Constant Change: Exploring Dynamic Oxygen Evolution Reaction Catalysis and Material Transformations in Strontium Zinc Iridate Perovskite in Acid. *J. Am. Chem. Soc.* **2021**, *143*, 9961-9971.
5. Chen, Y.; Li, H.; Wang, J.; Du, Y.; Xi, S.; Sun, Y.; Xu, Z. J. Exceptionally active iridium evolved from a pseudo-cubic perovskite for oxygen evolution in acid. *Nat. Commun.* **2019**, *10,* 1-10.
6. Grimaud, A.; Demortière, A.; Saubanère, M.; Dachraoui, W.; Duchamp, M.; Doublet, M.; Tarascon, J.M. Activation of surface oxygen sites on an iridium-based model catalyst for the oxygen evolution reaction. *Nat. Energy* **2016**, *2*, 1–10.
7. Seitz, L.C.; Dickens, C.F.; Nishio, K.; Hikita, Y.; Montoya, J.; Doyle, A.; Kirk, C.; Vojvodic, A.; Hwang, H.Y.; Norskov, J.K. A highly active and stable $IrO_x/SrIrO_3$ catalyst for the oxygen evolution reaction. *Science* **2016**, *353*, 1011–1014.
8. Shang, C.; Cao, C.; Yu, D.; Yan, Y.; Lin, Y.; Li, H.; Zeng, J. Electron correlations engineer catalytic activity of pyrochlore iridates for acidic water oxidation. *Adv. Mater.* **2019**, *31,* 1-6.
9. Yang, L.; Chen, H.; Shi, L.; Li, X.; Chu, X.; Chen, W.; Li, N.; Zou, X. Enhanced iridium mass activity of 6h-phase, ir-based perovskite with nonprecious incorporation for acidic oxygen evolution electrocatalysis. *ACS Appl. Mater. Interfaces* **2019**, *11*, 42006–42013.
10. Chen, H.; Shi, L.; Liang, X.; Wang, L.; Asefa, T.; Zou, X. Optimization of Active Sites via Crystal Phase, Composition, and Morphology for Efficient Low-Iridium Oxygen Evolution Catalysts. *Angew. Chem. Int. Ed.* **2020**, *59,* 19654-19658.
11. Liang, X.; Shi, L.; Cao, R.; Wan, G.; Yan, W.; Chen, H.; Zou, X. Perovskite-Type Solid Solution Nano-Electrocatalysts Enable Simultaneously Enhanced Activity and Stability for Oxygen Evolution. *Adv. Mater.* **2020**, *32,* 1-8.
12. Miao, X.; Zhang, L.; Wu, L.; Hu, Z.; Shi, L.; Zhou, S. Quadruple perovskite ruthenate as a highly efficient catalyst for acidic water oxidation. *Nat. Commun.* **2019**, *10,* 1-7.

Disclaimer/Publisher's Note: The statements, opinions and data contained in all publications are solely those of the individual author(s) and contributor(s) and not of MDPI and/or the editor(s). MDPI and/or the editor(s) disclaim responsibility for any injury to people or property resulting from any ideas, methods, instructions or products referred to in the content.
